# A Novel Approach for the Characterization of the Textural Properties of Table Olives: Acoustic Compression Related to Sensory Analysis

**DOI:** 10.3390/foods12020241

**Published:** 2023-01-05

**Authors:** Martina Bacceli, Nicola Simone, Barbara Lanza, Angelo Cichelli

**Affiliations:** 1 Council for Agricultural Research and Economics (CREA), Research Centre for Engineering and Agro-Food Processing (CREA-IT), Via Nazionale 38, I-65012 Cepagatti, Italy; 2 Department of Innovative Technologies in Medicine and Dentistry (DTIMO), University “G. d’Annunzio” of Chieti-Pescara, I-66100 Chieti, Italy

**Keywords:** crunchiness, sensory analysis, table olives, texture analysis

## Abstract

This research was performed on marketed table olives. We investigated possible correlations among textural parameters obtained using both sensory assessment and instrumental textural analysis. The purpose of this research study was to find out any possible correlation between the two different analysis methods, especially in relation to acoustic compression. Up to now, there are no available studies on this topic. Samples from different olive cultivars and different processing methods were analysed, and a data matrix resulting from four textural/acoustic and six sensorial kinaesthetic parameters was processed. The two parameters “S_crunch” and “T_noise” (the “S” letter is for “sensorial”, and the “T” letter is for “textural”) showed complementarity, but they did not discriminate properly. The textural values of “T_flesh_h” and the sensory values of “S_flesh_h” were directly correlated to “S_crunch”, and as an unexpected result, the textural values of “T_skin_bs” and the sensory values of “S_skin_h” were closely linked to each other. Regarding the analysed parameters, the results showed that the two techniques are clearly complementary and could constitute a valid tool for varietal characterization and for determining the instrumental and organoleptic qualities of the product; it was not possible to proceed with the characterization by type of processing method, as the dataset was not large enough.

## 1. Introduction

The quality of a food is nowadays described by ISO 9000: 2015 as the “degree to which a set of inherent characteristics of an object fulfils requirements” [1].

The evaluation of sensory properties has long been accompanied by the analysis of the kinaesthetic characteristics of food; it is performed with increasingly sophisticated texture analysers in order to obtain a final product that results to be pleasant for consumers. Thus, we may say that food quality is evaluated by consumers through its textural properties [2]. The sensory properties of a food are among the main characteristics for defining the global quality, and they represent the features most appreciated by consumers. 

The contribution of texture to consumer satisfaction with a food product has been studied a lot in recent decades, and studies indicate that the texture analysis profile of a food is mainly related to its crispness [3,4] and crunchiness [4]. Therefore, instrumental and sensory analyses are two different ways to study the right crispness/crunchiness of food palatability, as well as other kinaesthetic properties. 

Crispness and crunchiness are textural attributes that are commonly associated with the freshness and firmness of foods. Crispness is a parameter evaluated by a person and assessed as the resistance occurring while pressing with incisors, while crunchiness is perceived by chewing with molars [4]. Borrowing terms from the physics of engineering, it is more appropriate to define “crispness” as the result of a force applied to cut the food (for example, incisors) and “crunchiness” as the result of a force applied to fracture the food by means of compression alone (for example, molars), according to the definitions given by many authors [5,6,7,8]. 

Crispness is a salient textural property of most fresh and dry food products. Crunchiness is related to hydrated tissues and their turgidity. Its loss, due to the adsorption of moisture and subsequent cell membrane disruption in tissues, is a major cause of rejection by consumers. Both crispness and crunchiness are related to food structure and its mechanical properties in terms of the capability to generate appropriate and different sounds during mastication and to dampen or amplify these sounds [9]. 

Another important textural parameter to be considered is chewiness. It is a highly destructive process that is based on the repeated application of a mechanical force exerted by the mandible with different types of teeth. We thus have a cutting force (exerted with incisors), a crushing force (exerted with molars) and so on, up to the transformation of food into a bolus. Crispness and crunchiness are described as a combination of kinaesthetic and auditory components, so it is not surprising that instrumental methods are developed to evaluate them by focusing on the measurements of these properties singularly or in combination [4]. 

That is why mechanical properties are investigated to assess the structural properties of materials; to measure resistance to compression, a texture analyser equipped with a probe that presses the tissues and an acoustic device that records the sounds produced is used.

After an in-depth literature search, it appears that there are no studies on the acoustic properties of table olives, even if there are some on specific textural features, such as peel hardness [10,11,12,13,14]. Texture evaluation is also possible using sensory tests. Sensory analysis alone is widely used in table olives, thanks to the official method for the organoleptic analysis of table olives that was validated by IOC (International Olive Council) in 2008 and then revised in 2021 [15]. This method is applied to the fruits of the domestic olive tree (*Olea europaea* L.) marketed following adequate treatment for direct consumption as table olives, in compliance with Commercial Standard [16]. Even if textural analysis has already been applied in a few studies on table olives, no one has tried to relate sensory kinaesthetic parameters to textural kinaesthetic parameters, so the aim of this research is to investigate “if” and “how” these correlations exist.

The characteristic sounds made by crunchy food being broken or crushed are due to the fracture of the cell walls. These acoustic emissions were used to quantify the crunchiness perceived, assuming that mechanical properties are strongly related to the acoustic ones. In fact, this research study was developed in order to identify any correlations between the textural parameters resulting from the sensory analysis of table olives and the textural parameters detected using an instrument, with the purpose to find out a constant synergy between the two methods and to use both to characterize table olives based on their cultivar or the transformation method used. 

Succeeding in this operation could help industries to create high-preference products; it could provide customers and quality control authorities with an effective method to better assess the quality of a product; last but not least, it could help scholars in future food quality research.

## 2. Materials and Methods

### 2.1. Sampling

For our experimental design, we looked for every marketed table olive product that clearly showed the cultivar on the label. This was to reduce all the textural variables derived from shape and dimension of the different cultivars; we only found a few products showing this information, so we took all the available products. Twenty-three different table olive samples from different olive cultivars and different processing methods (Table 1) were processed. The four selected cultivars are the most representative ones in Italy. Concerning the processing methods, the most used ones were the following three: the “Castelvetrano”, “Sevillan” and “Greek” methods. The “Castelvetrano” method is typically used for “Nocellara del Belice” cv., and the “Greek” method is typical for “Taggiasca”; for “Itrana” cv., the “Bella di Cerignola”, “Sevillan” and “Greek” methods are the most common ones [17]. All samples were labelled with a unique code as shown in Table 1.

### 2.2. Sensory Analysis

The sensory evaluation of table olives was performed by a group of 8–10 expert tasters selected according to personal attitudes and led by a panel leader, forming the resident official tasting panel of table olives of CREA-IT “Council for Agricultural Research and Economics—Research Centre for Engineering and Agro-food Processing” sited in Pescara. The sensory analysis was carried out in a tasting room set according to standard COI/T.20/Doc. No. 6 “*Guide for the installation of a test room*” [18]. The sample of table olives for analysis was presented in standard tasting glasses (COI/T.20/Doc. No. 5 “*Glass for oil tasting*” [19]). A glass contained as many olives as the bottom of the glass could hold when the olives were placed side by side in a single layer. When brined table olives were analysed, sufficient covering liquid was poured over the olives to cover them fully. When the olives were above the 91/100 size grade, the volume of sample contained in the glass was never more than half the height of the glass (i.e., 30 mm). In the case of table olives belonging to a size grade below 91/100, the sample for testing in the glass comprised no less than three olives. When brined table olives were analysed, the quantity of covering liquid in the glass came up to at least three-quarters of the height of the olives. The glass was covered with the attendant watch glass. The samples of table olives intended for tasting were kept in glasses at ambient temperature, between 20 and 25 °C, under white light (daylight). Samples were assigned a code comprising digits, letters or both, which was marked using odourless markers. 

**Figure 1:**
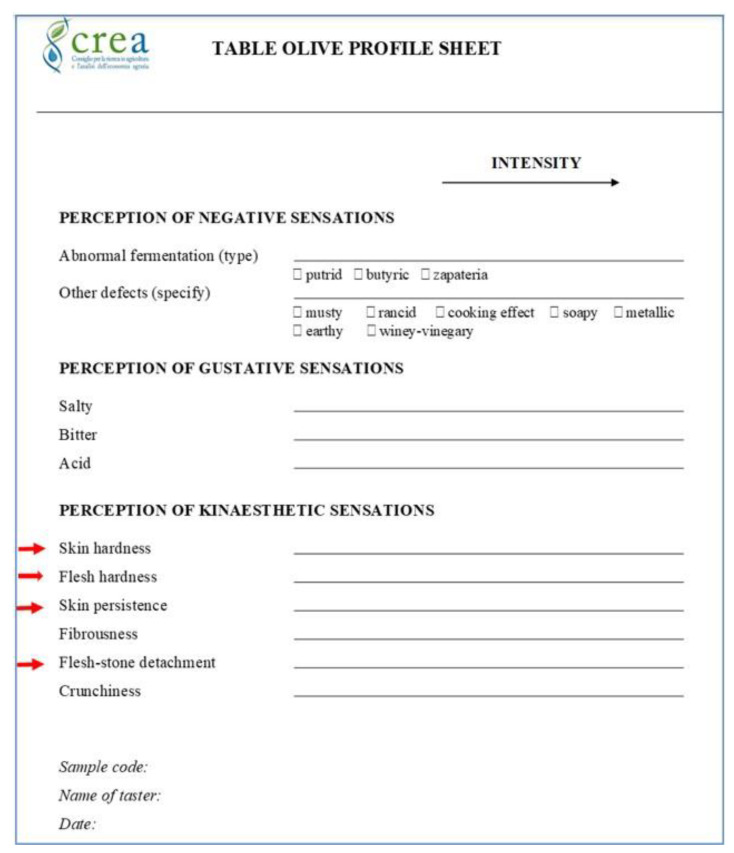
Experimental profile sheet based on the IOC official profile sheet (COI/OT/MO No. 1/Rev. 3—June 2021) [15].

For this study, an experimental profile sheet obtained by slightly modifying the IOC official sheet [15], as highlighted by red arrows in Figure 1, was utilized.

As shown, “hardness”, already reported in the original IOC sheet, was split into “skin hardness” and “flesh hardness”. The other descriptors included were “skin persistence” and “flesh-stone detachment”. The descriptors of “crunchiness” and “fibrousness” remained unchanged compared to the official profile sheet.

“Skin hardness” is assessed by initially placing an olive between the incisors to evaluate resistance to cut and penetration. “Flesh hardness” is assessed by chewing with the molar teeth, perceiving the resistance to deformation. Olive skin, due to its peculiar tissue composition, rich in waxes, is a tissue with greater cohesion and resistance than the underlying flesh (the mesocarp), so it can persist in large fragments during the chewing action. The waxes inside the skin make it resistant to the compression exerted by molars and premolars. In addition, some varieties have a thicker and leatherier cuticle than others; therefore, the attribute of “skin persistence” is assessed with the duration and intensity of repeated chewing until the complete destruction of the cuticle and is to be considered a characterizing parameter. “Flesh-stone detachment” is the parameter that evaluates how easily the stone separates from the flesh inside the oral cavity under the action of incisors and molars and how “clean” from pulp residues the stone is after expelling it from the mouth. 

We also added some boxes in the negative sensation section specifying all the abnormal fermentations and the defects to help tasters to recognize them.

New descriptors were identified and approved after the classic procedural process for the drafting of a sensory evaluation sheet, which consists of the following:

- Bibliographic research on works already carried out;
- Development of a specific dictionary;
- Group activities aimed at identifying all the sensory attributes that may affect the product;
- Elimination of generic and/or redundant attributes;
- Validation of the remaining attributes and their detailed description;
- Elimination of attributes not directly relevant to the purpose of the research.

### 2.3. Texture Analysis

The instrument used for textural analysis was TA.XT_PlusC ™ (Stable Micro Systems Ltd., Godalming, UK). The load cell used had a maximum operating range of 10 kg. The probe used for the measurements had a 10 mm diameter (P/10) [20]. The detected signal was transmitted to a PC through special proprietary software (Texture Exponent Connect 32 bit). The signal received was processed and represented graphically in the form of a force/time curve, which also displayed the acquisition of the various parameters set before the experiment. A specific macro was used in this set of measurements. Before testing each batch of samples, the height was calibrated to be able to always perform the test starting from the same position; a contact force of 100 g and a test speed of 2 mm/s were used. The microphone (Brüel Kjaer; Type 2671 Naerum; Denmark) included an “Acoustic Envelope Detector” (AED), which allowed us to exclude the background sounds emitted by the texture analyser and had high sensitivity to the frequencies emitted by crunchy products. The microphone was calibrated with an acoustic calibrator, type 4231 (1 Hz; Brüel Kjaer), setting the amplifier to a potentiometric value of 4/11; gain was then kept constant for all samples. The position of the microphone was 1 cm away from the sample at 45°.

The textural analysis was conducted on 10 olives for each sample. The test we used was a “compression” type test that was performed until the cuticle broke and the probe penetrated the flesh. The breaking phenomenon of the cuticle created the sound perceived by the microphone that was then measured. In our case, the device acquired 10 measurements per sample and built the force/time curve overlapping the individual acquisitions. The parameters considered were the skin breaking strength expressed in grams, the skin breaking time expressed in seconds, the noise produced during compression in decibels and the flesh hardness expressed in grams.

**Figure 2:**
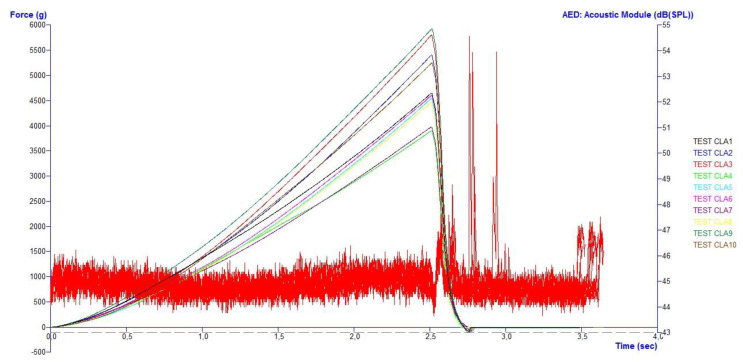
A representative compression/acoustic plot.

A representative compression/acoustic plot is shown in Figure 2.

### 2.4. Data Analysis

A descriptive statistical analysis of the results obtained with sensory evaluations (median, robust variance coefficient % and robust standard deviation) was conducted using the specific statistic program provided in Method (COI/OT/MO/Doc.1/Rev.3 Annex 3: Sensory analysis of table olives-computer program) [15] to obtain the necessary values for the definition of sensory profiles.

The data processing (Shapiro–Wilk normality test, Spearman’s correlation tests, PCA and cluster analysis) of the matrix was conducted using the free software PAleontological STatistics Version 4.10 (Øyvind Hammer, Natural History Museum, University of Oslo).

This matrix resulted from 4 textural/acoustic + 6 sensory parameters, and it consisted of 23 different batches of samples. Spearman’s correlation was performed with data transformed into ranks. The PCA analysis was performed on normalized data. The cluster analysis had the following parameters: aggregative analysis (bottom-up), Ward’s algorithm, Euclidean distance and non-constrained data.

In Table 2, all the variables that were considered, along with their descriptions and their units of measure, are shown.

## 3. Results

**Figure 3:**
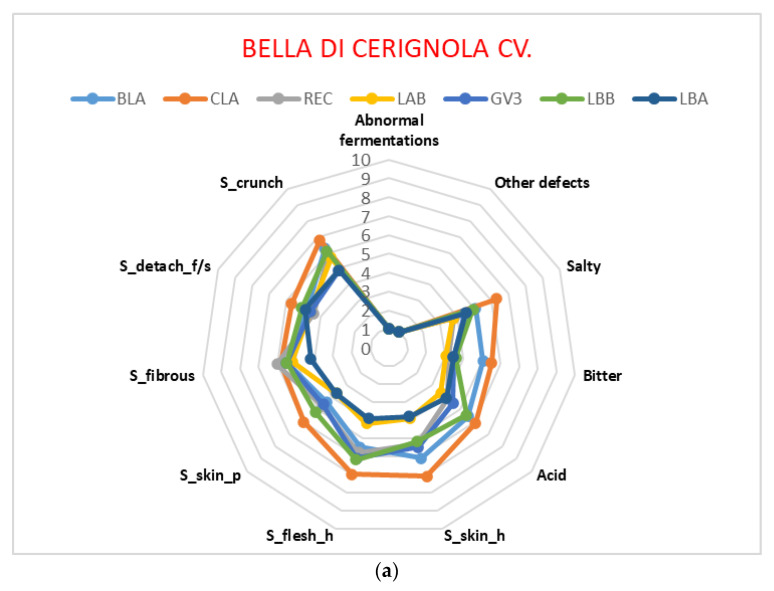

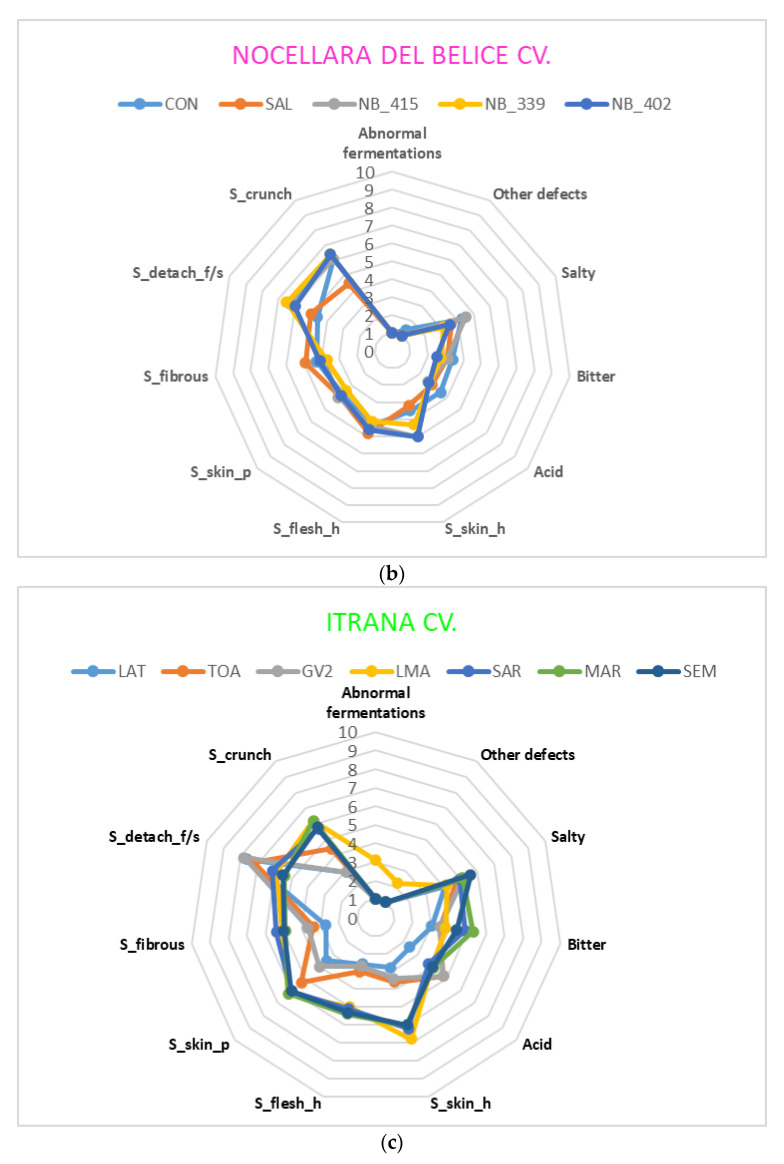

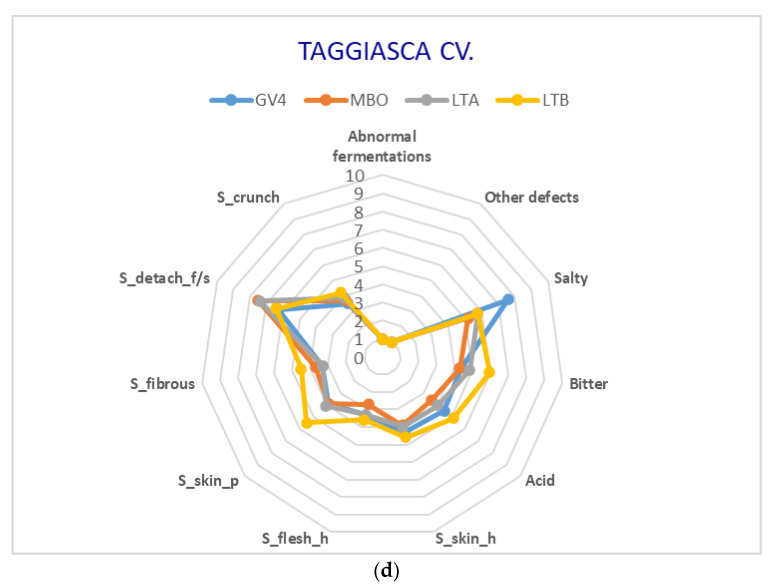
Sensory profiles of samples. (**a**) Bella di Cerignola cv. sensory profiles. (**b**) Nocellara del Belice cv. sensory profiles. (**c**) Itrana cv. sensory profiles. (**d**) Taggiasca cv. sensory profiles.

Sensory profiles were obtained considering all the 11 parameters assessed in the evaluation sheet and are presented in the form of radar graphs, cumulative for each cultivar (Figure 3). All the parameters present in the profile sheet were assessed and thus reported in radar graphs. “Bella di Cerignola” (Figure 3a) samples were quite balanced in their complete profile, especially concerning textural properties; CLA showed the highest values, while LBA and LAB had the lowest ones. The widest ranges were found for flesh hardness and skin hardness. In the case of “Nocellara del Belice” cv. (Figure 3b), all five samples had rather low median values of “skin hardness” and “flesh hardness”, while the “crunchiness” parameter of two of those samples (NB_339 and NB_402) assumed high values, second only to the “Bella di Cerignola” CLA sample. The “Itrana” cv. graph (Figure 3c) shows more complexity, due to the different ripening stages of the samples; three of them were turning black, and four were green. The ripening stage has a great influence on the cell structure of olive flesh and skin, so we expected a difference in the textural parameters; thus, we could observe, in the black-turning “Itrana” olives (LAT, TOA and GV2), the lowest values of some textural parameters, such as flesh hardness (2.6), fibrousness (2.7) and skin hardness (2.8). Similar values could be observed in the “Taggiasca” cv. samples, which were completely black and ripe (Figure 3d). Moreover, the “Taggiasca” samples also showed very low values of all the other parameters considered. This is understandable, given the small size of the olives, the black ripening stage and the transformation process applied to this cultivar [17].

From the sensory profiles, the six parameters related to the kinaesthetic properties were used to build a matrix together with the textural data obtained with the instrument. The considered matrix, as described in paragraph 2.4, was treated at several levels. First, the normality and homoskedasticity of the obtained data were verified to better understand which statistical tests were more appropriate for further detailed data analyses. By performing the preliminary tests, we observed that most of the variables were normally distributed, except for the parameters “T_noise”, “S_skin p” and “S_crunch”, which rejected the null hypothesis (Table 3). According to these results, we then proceeded to carry out non-parametric tests.

**Figure 4:**
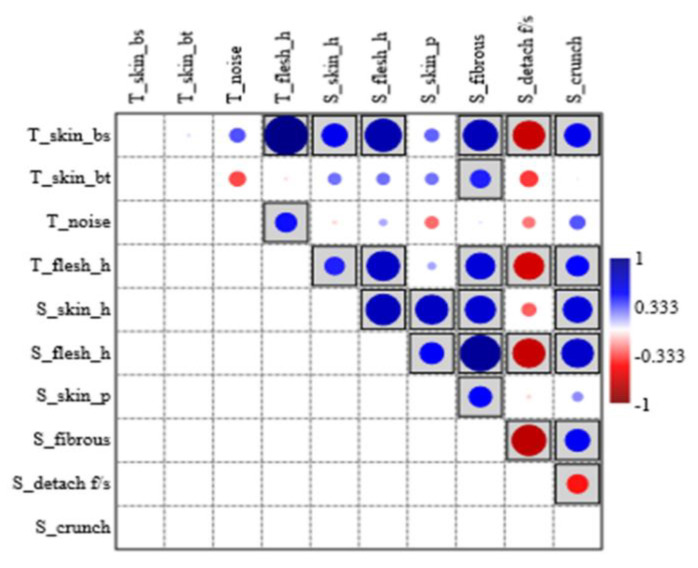
Plot of Spearman’s rank correlations.

To verify if some correlations existed (and to quantify them), “Spearman’s rank correlation” was preferred due to its “robustness” and due to the different nature of the data. In Figure 4, the plot obtained shows all the resulting correlations among sensory and textural parameters. The blue dots represent positive linear correlations, and the red ones display negative linear correlations; the intensity of the colour and the size of the dots indicate the strength of the correlation. Moreover, the grey boxes around the dots indicate all the statistically significant *p*-values. In Table 4, the r_s_ values and the related *p*-values are shown.

From the data obtained with “Spearman’s rank correlation”, we could assume that there were numerous correlations; unfortunately, the “T_noise” variable was only statistically correlated with a textural parameter, “T_flesh_h”, as expected. On the other hand, it is interesting to note the strong statistical links between “T_flesh_h” and the sensory descriptors “S_crunch”, “S_skin_h”, “S_flesh_h” and “S_fibrous”; there was also a negative linear correlation between “T_flesh_h” and “S_detach_f/s”. All these results allowed us to validate the effectiveness of our experimental profile sheet. To confirm this, we can point out another relevant correlation, the one between “T_skin_bs” and the sensory parameters “S_skin_h”, “S_flesh_h”, “S_fibrous”, “S_crunch” and “S_detach_f/s”. Furthermore, there were also many correlations among sensory parameters, such as “S_skin_h” and “S_flesh_h”; “S_skin_h” and “S_skin_p”; “S_fibrous” and “S_flesh_h”(high correlation value); and “S_crunch”, and “S_skin_h”, “S_flesh_h”, “S_fibrous” and “S_detach_f/s”. This evidence was expected on a logical basis, and it was confirmed with statistical analysis. Beyond these peculiar values, however, there were many other correlations among the different parameters, which suggests that the scope of the research was well defined.

**Figure 5:**
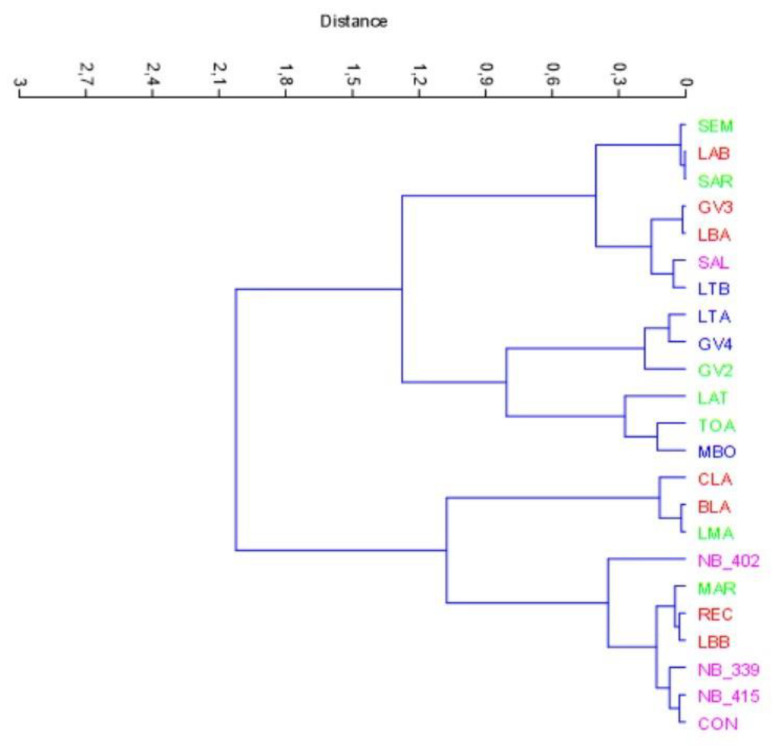
Cluster analysis (aggregative method, Ward’s algorithm, not constrained and Euclidean distance) performed on variables “S_crunch” and “T_noise”. Legend: green, Itrana cv.; blue, Taggiasca cv.; lilac, Nocellara del Belice cv.; red, Bella di Cerignola cv.

After that, a first aggregative cluster analysis was carried out by only taking into consideration the “S_crunch” and “T_noise” parameters (Figure 5). The goal was to understand if these two parameters alone could somehow characterize the different cultivars. Given the simplicity of the matrix composed of only two variables, Ward’s algorithm was set to create a cluster based on the shortest distance between two groups and without using the “constrained” parameter. The results obtained are interesting and show quite evident similarities within the groups of the different varieties analysed, with some easily explained exceptions. 

In Figure 5, we can immediately detect a varietal group, Nocellara del Belice cv., which clearly diverges from all the others by placing itself in a compact section of the graph, except for one outlier (SAL), which is closer to one Taggiasca cv. sample (LTB). This is because the SAL sample obtained rather low values of both sensory crunchiness and perceived textural noise, so it is clustered next to more similar values of LTB. “Taggiasca” cv., in fact, had generally lower values related to crunchiness and noise produced, and this was due to the nature of the olive fruit and the transformation process used. Nocellara del Belice cv. was confirmed, in any case, to be the best differentiated varietal group in terms of similarities and clearly stands out from the other groups in a separate cluster with three out of five samples (NB_415, NB_339 and CON) and a fourth sample (NB_402) with an intercultivar similarity of about 0.4. The other groups of cultivars are very fragmented in the graph, so we could not identify other homogeneous groups in our sample. We can see the high variability of Itrana cv. for the two considered parameters; sometimes, it is closer to Bella di Cerignola cv. (harder and noisier), and sometimes, it is closer to Taggiasca cv. (softer and less noisy).

**Figure 6:**
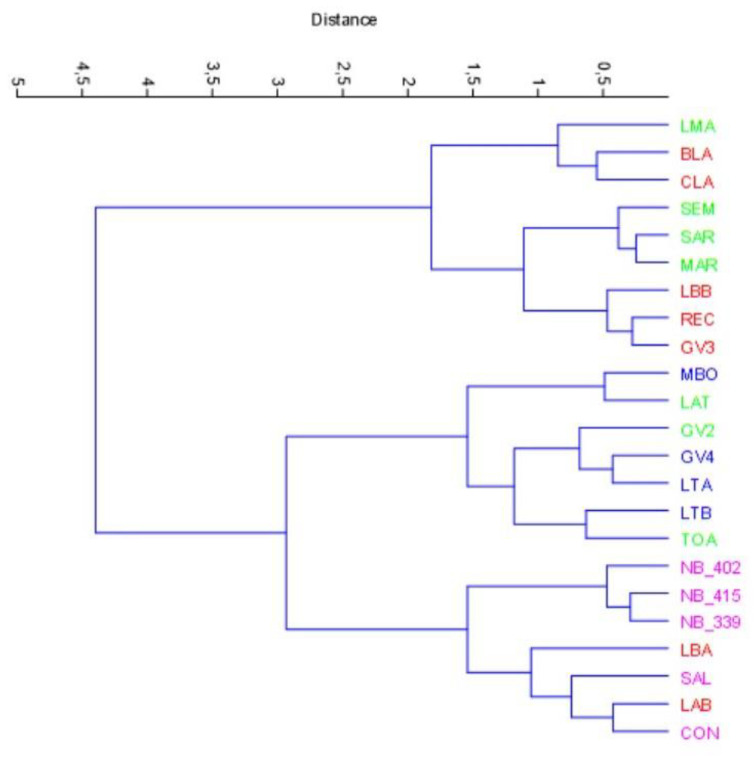
Cluster analysis (aggregative method, Ward’s algorithm, not constrained and Euclidean distance) of the whole data matrix. Legend: green, Itrana cv.; blue, Taggiasca cv.; lilac, Nocellara del Belice cv.; red, Bella di Cerignola cv.

We applied a cluster analysis to the entire matrix of textural and sensory parameters, and the results are shown in Figure 6. 

In the graph (Figure 6), we can distinguish three main different clusters. One of these is represented by the Nocellara del Belice group, which clusters in a group of three elements with similarity close to 0.5; this tiny group clusters with similarity values close 1.5 with a mixed group made of Nocellara del Belice/Bella di Cerignola. The other cluster we can observe is the Taggiasca group enclosed in an enclave within the Itrana group at the black ripening stage (GV2, LAT and TOA) with values of similarity of around 1.5. The last cluster is composed of Bella di Cerignola and Itrana at the green ripening stage processed as “Itrana Bianca” products (MAR, SAR, SEM and LMA) with similarity values of around 1.8. These results could be related to the same ripening stage that affects the kinaesthetic characteristics.

It was decided to further delve into the data obtained by analysing the entire matrix using the PCA methodology to understand which components assumed greater weight in the discrimination between the different groups of samples. 

**Figure 7:**
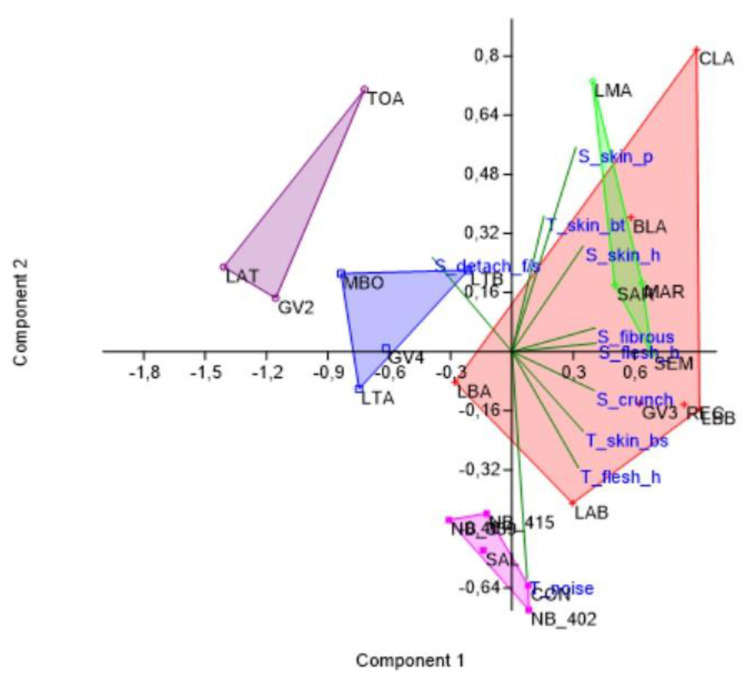
PCA on the entire data matrix. Legend: green, Itrana cv.; purple, black Itrana cv.; blue, Taggiasca cv.; lilac, Nocellara del Belice cv.; red, Bella di Cerignola cv.

As can be seen in Figure 7, there are four well-differentiated groups of samples. One group of samples (Nocellara del Belice) clearly constitutes a separate set, with the “T_noise” parameter heavily contributing to this differentiation from the other convex hulls. 

As already pointed out by the cluster analysis results, Itrana cv. is split in two different groups according to the ripening stage; samples at the black ripening stage are closer to Taggiasca cv., while green Itrana are in the Bella di Cerignola group. 

## 4. Discussion

In the literature, the topics regarding the importance of textural and sensory properties in food quality evaluations [21,22,23] and the differences between crunchiness and crispness [4,24,25,26,27,28] have been extensively discussed. 

On the other hand, few studies are available relating the textural analysis of table olives. Some research focused on textural properties applied to the shelf life of table olive packaging [5,8], while the textural modification occurring in table olives when chewed was assessed from a microstructural point of view [7] by analysing the fracture surface with scanning electron microscopy. Another study linked textural properties and antioxidant activity in naturally fermented green olives during their processing [6] with different NaCl concentrations in brine. From these studies, the problem of textural characterization emerges clearly, especially due to the extreme variability of table olive products, which need to be distinguished according to both cultivars and transformation methods. 

At the beginning of our investigation, we were conscious of the difficulty in perceiving the crunchiness on table olives because of their moisture. So, we had to look at similar studies on other foods. An interesting work on fresh-cut apples shows the influence of the maturity degree on textural properties [2], which fits our purposes in some ways. They tested the textural properties with a similar process, using the parameters “displacement” (mm) and “sound pressure level” (dB) with positive results. In fact, the sum of parameters they used could be considered as crispness. For table olives, it would be better to use the terms crunchiness rather than crispness because of the transformation process that affects tissue composition and also because they are mostly crushed with molar teeth rather than incisors.

Even only considering the official method of sensory analysis, regarding table olives, in the literature, no research studies on their crunchiness can be found. The method itself gives as “standard values” for the table olive crunchiness scale a median value of 2.5 to peach in syrup and a median value of 10 to celery stalk. Yet, no other studies on olive crunchiness are present. This gap has to be filled, and we tried to start a brand-new branch of research on table olive characterization. 

According to the obtained and analysed data, textural analysis and sensory analysis seem to share connections, and they are able to discriminate and characterize a set of samples in terms of different cultivars and different transformation processes. Despite the relatively small number of samples (23) from four cultivars (7 + 7 + 5 + 4), the results obtained using only the parameters “S_crunch” and “T_noise” are satisfactory and indicative, but nevertheless not crucial; in fact, if the “noise produced” parameter is considered individually, it fails to characterize “crunchiness”, but it plays an important role in the sum of the variables (as per the results of the cluster analysis and PCA). 

If we then consider the whole set of parameters analysed, both at the textural and sensorial levels, the results appear to be much clearer, and we can conclude as follows:

(1) The two techniques are clearly complementary and can constitute a valid tool for varietal characterization and for determining the instrumental and organoleptic quality of the product.

(2) It was not possible to proceed with the characterization by type of processing method, as the dataset was not large enough to do so.

(3) As an expected result, the textural values of “T_flesh_h” and the sensory values of “S_flesh_h” were directly correlated to “S_crunch”, yet it is considered that correlation does not mean causation.

(4) As an unexpected result, the textural values of “T_skin_bs” and the sensory values of “S_skin_h” were directly correlated to “S_crunch”, yet it is considered that correlation does not mean causation.

(5) The two parameters “S_crunch” and “T_noise” showed some kind of complementarity, but they did not seem sufficient to discriminate properly.

(6) The crunchiest cv. resulted to be Nocellara del Belice, and this can explain why this is a cultivar with great consumer appreciation. Moreover, the “Castelvetrano” processing method is quick (few days vs. months for “Greek” and “Sevillan”), and this can also explain the high turgidity of the olives.

## 5. Conclusions

In conclusion, this coupled method is indeed a powerful and useful tool to obtain a table olive “kinaesthetic profile” that is as complete as possible. Of course, a much greater number of sample datasets is required to increase the possibility of a perfectly normal distribution of data and thus to better apply the available parametric tests, which are more powerful than non-parametric ones. This could result in a deeper comprehension of the obtained data and the potential discovering of new correlations. Moreover, two different datasets of specimens from the same cv. processed with different methods could provide precious information on the nature of olive crunchiness and the influence of the transformation process used. In relation to what is available in the literature, this work of comparison between sensory and textural analyses supplements those already available (on other products), which have been conducted for several years due to the relevance assumed by this topic for commercial purposes; however, little research has been performed on table olives, and there is no focus on the “crunchiness” parameter of these products. Thus, the present work turns out to be a pioneering study in the field of table olives on the topic considered. Our future studies will include a database of sensory crunchiness for each olive cultivar used for table olive production and for each different processing method, involving different panels. This could be performed quite easily due to the international standardization of the sensory assessment method. On the other hand, the same should be conducted using textural analysis, but only few labs are equipped with suitable instruments. Eventually, the two databases will be merged to create a sort of “fingerprint” of table olive crunchiness.

## Figures and Tables

**Table 1:** Labelling, processing methods and ripening stages of the twenty-three samples distinguished by cv.

Label Code	Cultivar	Processing Method	Ripening Stage
BLA	Bella di Cerignola	Greek style	Green
CLA	Bella di Cerignola	Greek style	Green
REC	Bella di Cerignola	Sevillan style	Green
LAB	Bella di Cerignola	Sevillan style	Green
GV3	Bella di Cerignola	Sevillan style	Green
LBB	Bella di Cerignola	Sevillan style	Green
LBA	Bella di Cerignola	Sevillan style	Green
LAT	Itrana	Greek style	Black
TOA	Itrana	Greek style	Black
GV2	Itrana	Greek style	Black
LMA	Itrana	Greek style	Green
SAR	Itrana	Greek style	Green
MAR	Itrana	Greek style	Green
SEM	Itrana	Greek style	Green
CON	Nocellara del Belice	Castelvetrano style	Green
SAL	Nocellara del Belice	Castelvetrano style	Green
NB_415	Nocellara del Belice	Castelvetrano style	Green
NB_339	Nocellara del Belice	Castelvetrano style	Green
NB_402	Nocellara del Belice	Castelvetrano style	Green
GV4	Taggiasca	Greek style	Black
MBO	Taggiasca	Greek style	Black
LTA	Taggiasca	Greek style	Black
LTB	Taggiasca	Greek style	Black

**Table 2:** Description of the variables.

Variable Code	Description	Unit of Measure
T_skin_bs	Skin breaking strength measured by texture analysis	g
T_skin_bt	Skin breaking time measured by texture analysis	sec
T_noise	Noise produced during compression measured by texture analysis	dB
T_flesh_H	Flesh hardness measured by texture analysis	g
S_skin_h	Skin hardness perceived by sensory analysis	Median value
S_flesh_h	Flesh hardness perceived by sensory analysis	Median value
S_skin_p	Skin persistence perceived by sensory analysis	Median value
S_fibrous	Fibrousness of the whole fruit perceived by sensory analysis	Median value
S_detach_f/s	Flesh/stone detachment perceived by sensory analysis	Median value
S_crunch	Crunchiness of the whole fruit perceived by sensory analysis	Median value

**Table 3:** Normality test of the variables.

Variable	Shapiro-Wilk
T_skin_bs	0.943
T_skin_bt	0.121
T_noise	<0.0001
T_flesh_H	0.743
S_skin_h	0.470
S_flesh_h	0.714
S_skin_p	0.005
S_fibrous	0.368
S_detach_f/s	0.123
S_crunch	0.016

**Table 4:** Table of Spearman’s rank correlation values split in two parts: The upper table shows the values of both positive (in blue) and negative (in red) significant correlations.

					Statistic					
	T_skin_bs	T_skin_bt	T_noise	T_flesh_h	S_skin_h	S_flesh_h	S_skin_p	S_fibrous	S_detach_f/s	S_crunch
T_skin_bs		0.06128	0.33992	0.96739	0.56924	0.81434	0.30739	0.76573	−0.71284	0.57638
T_skin_bt	0.06128		−0.35681	−0.06128	0.27752	0.28042	0.27573	0.44043	−0.38842	0.03294
T_noise	0.33992	−0.35681		0.47332	−0.07715	0.16316	−0.28756	0.04656	−0.26416	0.33226
T_flesh_h	0.96739	−0.06128	0.47332		0.43769	0.73424	0.17402	0.65181	−0.67079	0.49814
S_skin_h	0.56924	0.27752	−0.07715	0.43769		0.77035	0.72755	0.68121	−0.31097	0.64288
S_flesh_h	0.81434	0.28042	0.16316	0.73424	0.77035		0.52940	0.89913	−0.71980	0.71085
S_skin_p	0.30739	0.27573	−0.28756	0.17402	0.72755	0.52940		0.50472	−0.07967	0.22037
S_fibrous	0.76573	0.44043	0.04656	0.65181	0.68121	0.89913	0.50472		−0.76296	0.54927
S_detach_f/s	−0.71284	−0.38842	−0.26416	−0.67079	−0.31097	−0.71980	−0.07967	−0.76296		−0.46207
S_crunch	0.57638	0.03294	0.33226	0.49814	0.64288	0.71085	0.22037	0.54927	−0.46207	
p (uncorrect)										
	T_skin_bs	T_skin_bt	T_noise	T_flesh_H	S_skin_h	S_flesh_h	S_skin_p	S_fibrous	S_detach_f/s	S_crunch
T_skin_bs		0.78120	0.11252	5.30 × 10^−14^	0.00458	2.26 × 10^−6^	0.15364	2.06 × 10^−5^	0.00014	0.00399
T_skin_bt	0.78120		0.09466	0.78120	0.19982	0.19497	0.20286	0.03544	0.06701	0.88140
T_noise	0.11252	0.09466		0.02254	0.72642	0.45695	0.18336	0.83293	0.22322	0.12138
T_flesh_H	5.30 × 10^−14^	0.78120	0.02254		0.03673	6.65 × 10^−5^	0.42712	0.00075	0.00046	0.01556
S_skin_h	0.00458	0.19982	0.72642	0.03673		1.71 × 10^−5^	8.35 × 10^−5^	0.00035	0.14866	0.00094
S_flesh_h	2.26 × 10^−6^	0.19497	0.45695	6.65 × 10^−5^	1.71 × 10^−5^		0.00938	5.52 × 10^−9^	0.00011	0.00014
S_skin_p	0.15364	0.20286	0.18336	0.42712	8.35 × 10^−5^	0.00938		0.01404	0.71783	0.31229
S_fibrous	2.06 × 10^−5^	0.03544	0.83293	0.00075	0.00035	5.52 × 10^−9^	0.01404		2.30 × 10^−5^	0.00664
S_detach_f/s	0.00014	0.06701	0.22322	0.00046	0.14866	0.00011	0.71783	2.30 × 10^−5^		0.02643
S_crunch	0.00399	0.88140	0.12138	0.01556	0.00094	0.00014	0.31229	0.00664	0.02643	

## Data Availability

Data is contained within the article.
